# Epitope mapping by random peptide phage display reveals essential residues for vaccinia extracellular enveloped virion spread

**DOI:** 10.1186/1743-422X-9-217

**Published:** 2012-09-24

**Authors:** Yong He, Yonggang Wang, Evi B Struble, Pei Zhang, Soma Chowdhury, Jennifer L Reed, Michael Kennedy, Dorothy E Scott, Robert W Fisher

**Affiliations:** 1 Laboratory of Plasma Derivatives, Division of Hematology, Office of Blood Research and Review, Center for Biologics Evaluation and Research, Food and Drug Administration, FDA/CBER/OBRR/DH/LPD, HFM-345, 1401 Rockville Pike, Rockville, MD, 20852, USA

**Keywords:** Orthopoxviruses, Monoclonal antibody, B-cell epitope, Immunogen, Vaccinia, Phage display library

## Abstract

**Background:**

A33 is a type II integral membrane protein expressed on the extracellular enveloped form of vaccinia virus (VACV). Passive transfer of A33-directed monoclonal antibodies or vaccination with an A33 subunit vaccine confers protection against lethal poxvirus challenge in animal models. Homologs of A33 are highly conserved among members of the *Orthopoxvirus* genus and are potential candidates for inclusion in vaccines or assays targeting extracellular enveloped virus activity. One monoclonal antibody directed against VACV A33, MAb-1G10, has been shown to target a conformation-dependent epitope. Interestingly, while it recognizes VACV A33 as well as the corresponding variola homolog, it does not bind to the monkeypox homolog. In this study, we utilized a random phage display library to investigate the epitope recognized by MAb-1G10 that is critical for facilitating cell-to-cell spread of the vaccinia virus.

**Results:**

By screening with linear or conformational random phage libraries, we found that phages binding to MAb-1G10 display the consensus motif CEPLC, with a disulfide bond formed between two cysteine residues required for MAb-1G10 binding. Although the phage motif contained no linear sequences homologous to VACV A33, structure modeling and analysis suggested that residue D115 is important to form the minimal epitope core. A panel of point mutants expressing the ectodomain of A33 protein was generated and analyzed by either binding assays such as ELISA and immunoprecipitation or a functional assessment by blocking MAb-1G10 mediated comet inhibition in cell culture.

**Conclusions:**

These results confirm L118 as a component of the MAb-1G10 binding epitope, and further identify D115 as an essential residue. By defining the minimum conformational structure, as well as the conformational arrangement of a short peptide sequence recognized by MAb-1G10, these results introduce the possibility of designing small molecule mimetics that may interfere with the function of A33 *in vivo*. This information will also be useful for designing improved assays to evaluate the potency of monoclonal and polyclonal products that target A33 or A33-modulated EV dissemination.

## Background

Despite the eradication of naturally occurring smallpox, the licensed smallpox vaccine is still administered to military personnel and first responders due to the threat of bioterrorism [1], as well as to individuals with potential exposure to monkeypox. In February 2008, the Centers for Diseases Control and Prevention (CDC) disposed of the last of its 12 million doses of Dryvax, the licensed first generation smallpox vaccine grown on the skin of calves. A new vaccine, ACAM2000, was licensed by the Food and Drug Administration in 2007 as a replacement. ACAM2000 is a replication-competent vaccinia virus clone derived from Dryvax and manufactured in large scale mammalian cell cultures. Efficacy was determined in a number of animal models and found to be non-inferior to Dryvax in eliciting an immunological response; however ACAM2000 has a similar safety profile when compared to Dryvax and introduces a level of risk for a small subset of individuals [2]. These complications may be severe and life-threatening. Severe adverse events following vaccination may include eczema vaccinatum (EV) in patients with atopic dermatitis and certain other skin conditions, and progressive vaccinia (PV) in immunocompromised patients [3-6].

Vaccinia Immune Globulin Intravenous (Human) (VIGIV), a polyclonal antibody preparation manufactured from plasma of vaccinia-immunized donors, is the only licensed therapy for smallpox vaccine complications. While no placebo-controlled clinical trials were performed with the currently available VIGIV product, the use of similar products has historically decreased mortality, from 100% to 50% for PV, and from 30-40% to 3-4% for EV (reviewed in [7]). In severe cases very high repeated doses of VIGIV have been used and in the context of widespread vaccination, VIGIV supply could be limiting [6,8]. Enhancing the potency of licensed VIGIV is challenging in part because virus neutralizing assays for screening donor plasma are laborious, require live virus, and are subject to the variability typically encountered in biological assays. Binding assays to quantitate antibody levels are problematic in the absence of specific epitope binding information or in the context of polyclonal preparations that may contain a mixture of neutralizing and non-neutralizing antibodies, and are therefore typically supported by use of a plaque reduction neutralization assay [9]. Since immunogenicity is a critical consideration in vaccine development, structural understanding of critical viral protein epitopes would aid development of feasible assays capable of measuring important antibody specificities in donor plasma and VIGIV.

During the poxvirus infectious life cycle, approximately 1% of intracellular mature virions (IMV) are wrapped with additional membrane and exocytosed as extracellular enveloped virus (EEV) (reviewed in [10]). While IMV may mediate host-to-host transmission [11,12], EEV are thought to be uniquely responsible for rapid spread of virus *in vivo* and present an important antibody target. Antibody-mediated inhibition of EEV release from infected cells and blockade of EEV entry have been demonstrated [13-15]. Passive immunization is more effective in polyclonal antibody preparations containing higher EEV antibody titers [16], and anti-EEV monoclonals provide protection in a mouse vaccinia intranasal challenge model [17]. Vaccination with EEV proteins can also elicit a protective immune response [18]. Unfortunately, in immunized individuals anti-EEV titers vary considerably and may decline over time post-vaccination [19,20]. Anti-EEV antibody levels are also variable among different VIG products (M. Kennedy and R. Fisher, unpublished data) suggesting that potency gains might be realized by selecting plasma of donors with more robust responses to EEV neutralizing surface determinants. However, identification and characterization of EEV neutralizing determinants is still incomplete and assays to measure EEV neutralizing activity are subject to a high degree of variability.

The EEV envelope contains several viral proteins, including A56R [21,22], F13L [23,24], B5R [13,25], A36R [26], A34R [27,28], and A33R [29]. Among those, B5 [30] and A33 [31] proteins are known neutralization or viral spread inhibition targets associated with the EEV membrane and/or infected cells. The A33 protein appears to regulate EEV egress from cells and interacts with A36 to antagonize superinfection of neighboring cells, promoting more rapid long-distance dissemination [32-34]. Antibodies such as MAb-1G10 directed against A33 block comet formation *in vitro* and can protect against poxvirus challenge *in vivo* in passive transfer models [31,35-37].

MAb-1G10 was initially characterized as an A33-binding monoclonal antibody that could provide partial protection *in vivo* against an intranasal VACV-WR challenge in a mouse model, as well as block EV spread in cell culture [37]. Although a disconnect between protective efficacy and antibody affinity has been demonstrated for antibodies raised against A33 [35], A33 has been evaluated as part of an effort to identify epitopes which might be cross-protective against multiple pathogenic poxviruses [38]. This analysis showed that the β-mercaptoethanol sensitive MAb-1G10 epitope on vaccinia A33 was not present in the monkeypox A33 ortholog A35; the interpretation was that the MAb-1G10 binding epitope was conformational in nature. Binding of MAb-1G10 to the monkeypox A35 protein could be restored by single-residue exchanges at positions 117, 118, and 120 changing the monkeypox sequence to the vaccinia sequence. Based on this information, residues 117–120 were implicated as core residues forming the MAb-1G10 epitope. The importance of this region was reinforced by crystallographic data from a fragment of the ectodomain of A33 (residues 98–185) [39]. A dimeric, β-strand rich structural model of vaccinia A33 with structural similarity with C-type lectins was proposed. The described structure featured 5 β-strands and 2 α-helices stabilized by 2 intramolecular disulfide bonds (C100-C109 and C126-C180). Residues 117–120 were mapped to a surface-exposed edge on the proposed monomer structure, well removed from the dimer and proposed ligand-binding interfaces.

To provide additional characterization of the epitope involved in cell to cell spread of vaccinia, we considered whether additional residues might influence MAb-1G10 binding in the context of the vaccinia A33 protein. In this study, we screened a random peptide phage display library to find peptides specifically bound by MAb-1G10. A conformationally constrained consensus motif of seven residues was analyzed against available A33 sequence and structural information to generate an epitope model, which was tested and confirmed by an alanine site directed mutagenesis approach. The results demonstrated that the negatively charged D115 is required for MAb-1G10 binding, and helps establish the minimum epitope core for MAb-1G10 binding in the intact vaccinia A33 protein. Our data also confirm that residue L118 contributes to epitope formation, in agreement with previous observations. Our study shows that an unbiased mapping strategy utilizing random peptide display technology can effectively map linear and conformational epitopes involved in facilitating cell to cell spread of vaccinia. This work also expands understanding of an important orthopoxvirus epitope, which may be exploited to improve and inform therapies for vaccinia and potentially smallpox.

## Results

### Screening of random peptide libraries

**Figure 1:**
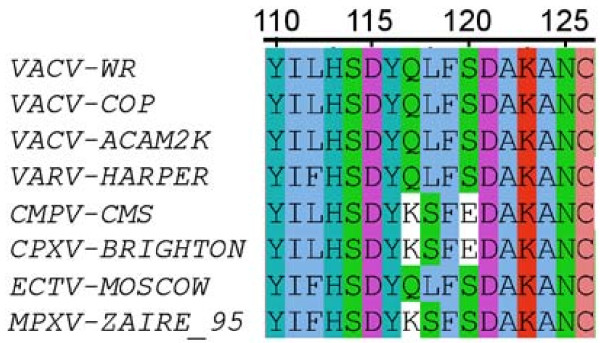
**Amino acid sequence alignment of orthopoxvirus A33 homologs.** D115 is conserved across species, while residues 118 and 120 demonstrate heterogeneity.

**Figure 2:**
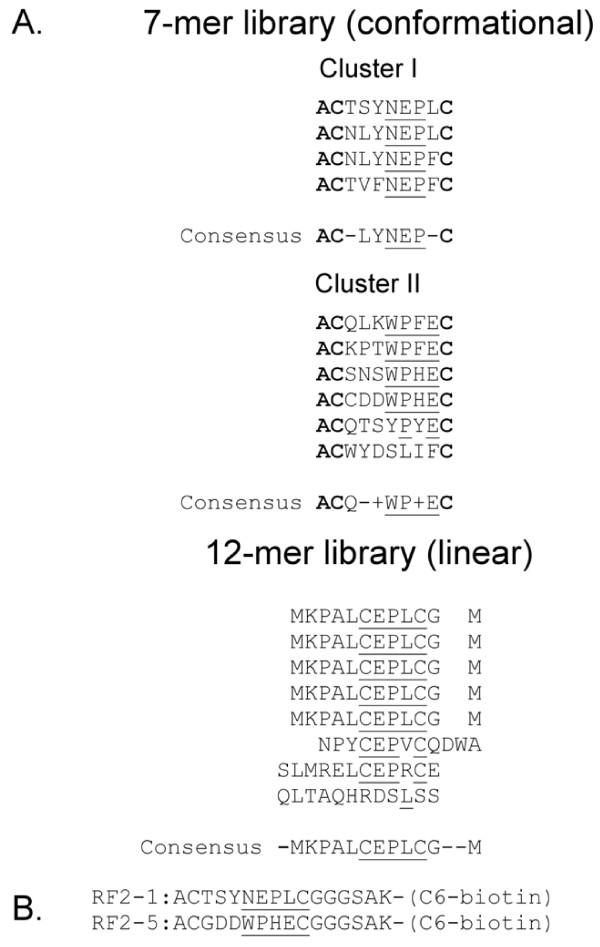
**Epitope mapping of MAb-1G10.** (**A**) Results of phage display screening against MAb-1G10 using a 7-mer (C7C; conformatially constrained by inclusion of flanking cysteines) and a linear (no conformational constraints) 12-mer library. Phage vector sequences are indicated by bold type. (**B**) Chemically synthesized epitope peptides containing Cluster I and Cluster II consensus sequences. Conserved residues are underlined.

**Figure 3:**
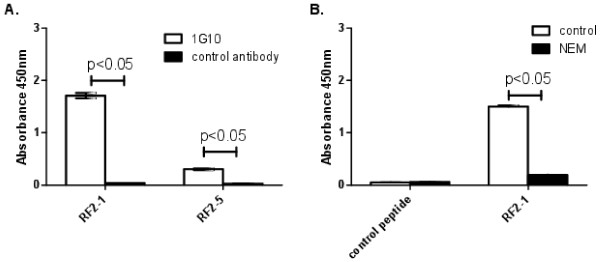
**MAb-1G10 binding to chemically synthesized peptides containing Cluster I and Cluster II consensus sequences.** (**A**) Peptides RF2-1 and RF2-5 demonstrate increased binding to MAb-1G10 when compared to an unrelated control antibody. (**B**) Treatment of reduced peptide RF2-1 with N-ethylmaleimide (NEM) significantly reduces MAb-1G10 binding compared to treatment with PBS alone (control), indicating the requirement for a conformational structure constrained by a disulphide bond. Binding levels were compared using Student’s t-test.

In considering the aligned sequences of poxvirus A33 homologs (Figure 1), we noted more subtle patterns of alternating highly-charged residues and hydrophobic stretches, and the striking heterogeneity of charged residues in the proposed region of the MAb-1G10 epitope. If non-convalent interactions among charged and hydrophobic residues influence regional conformation, then the context of the MAb-1G10 epitope (for example, presentation of the epitope in a monkeypox A35 versus vaccinia A33 backbone) might yield different epitope mapping information. On this basis we decided to pursue additional characterization of the MAb-1G10 epitope. To obtain unbiased information on the conformationally distinct epitope interacting with MAb-1G10, a disulfide-constrained heptapeptide library screening approach was used. In this approach, the randomized peptide segment is flanked by paired cysteines, which are oxidized during phage assembly to present the peptide as a taut loop at the N-terminus of the minor phage coat protein PIII. Ten MAb-1G10 binding peptides were isolated from the conformational library screening, none of which contain vaccinia virus A33 sequence (Figure 2A). Two consensus motifs were identified: CXXY(F)NEPL(F)C, and CXXXWPF(H)EC. Biotinylated peptide mimics were subsequently constructed to verify MAb-1G10 binding in a solid-phase assay (Figure 2B). Strong interaction of MAb-1G10 with one of the peptides (RF2-1), containing the CXXY(F)NEPL(F)C motif, was confirmed in the ELISA based assay (Figure 3A). We observed that ***N***-ethylmaleimide treatment of reduced peptide RF2-1 blocked MAb-1G10 binding (Figure 3B), suggesting that intact disulfide bonds were important for epitope conformation. A second pass of library screening was undertaken to determine if additional consensus motifs might be obtained. The second screen utilized a phage library in which linear dodecapeptides were presented at the N-terminus of phage coat protein PIII. Two MAb-1G10 binding peptides were obtained by screening the linear peptide library, neither of which contained viral sequence and both containing a consensus CEPLC motif (Figure 2A). A comparison of peptide sequences obtained from the 2 library screens suggested that the conformationally constrained CEPLC sequence was likely to be functionally identical to the minimal core MAb-1G10 epitope.

### Molecular modeling of the MAb-1G10 epitope

**Figure 4:**
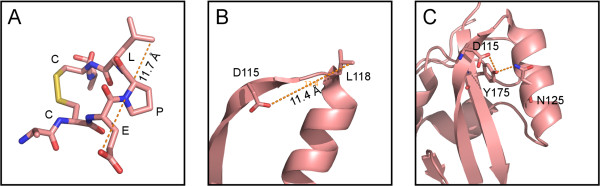
**Modeling of proposed MAb-1G10 binding structures.** (**A**) Model of the phage display peptide CEPLC in stick representation; disulphide bond is depicted in yellow, oxygen and nitrogen atoms are colored red and blue respectively. (**B**) Cartoon representations of the A33 structure using coordinates from [PDB: 3K7B] [39]. The strand-loop-helix region of the A33 protein similar to the phage display peptide CEPLC (see text) is shown. Leucine and aspartate side chains are depicted in stick representation and the distance between them displayed. (**C**) Alternative conformational epitope also consistent with phage display data where long-range interactions between A33 residues could be involved; mutational analysis did not support this hypothesis. Distances ~3 Å between polar groups are shown in broken lines.

While the CELPC sequence is not present in vaccinia A33, we reasoned that the conformation of the constrained CELPC motif might be identifiable in the folded A33 protein. A molecular model of the short consensus CELPC peptide was constructed (Figure 4A) as an aid in identifying potential MAb-1G10 binding regions in the intact A33 molecule. For this, the molecular coordinates of two loops with (pdbid 3DXQ, sequence C(177)DPLC(181)) and without (pdbid 1M6B, C(304)GGLC(308)) disulphide bonds were extracted. Although both structures are loops, the presence or absence of the disulphide bond prescribes a different topology for these sequences. To better visualize the difference, we “mutated” the CGGLC sequence *in silico* to the phage consensus sequence (CEPLC) using Pymol and subsequently undertook energy minimization of this model for 5000 steps in vacuum using CHARMM field. The resulting model showed the disulfide bonded CEPLC peptide featuring an 11.7 angstrom distance between the charged glutamate side chain and the hydrophobic leucine residue, whereas in the reduced loop the similar distance (aspartate-leucine) was only 6.9 angstroms. If this model was accurate, two outcomes were possible. First, reducing the disulphide bond would reduce the binding of the phage peptide to MAb-1G10 antibody by virtue of altering the conformation of the loop and second, and most importantly, the mature A33 molecule must contain in its surface residues similar to E and L at a similar relative position as the consensus phage peptide. Probing the published structure of A33 for a surface-exposed region with a distance between charged and hydrophobic residues similar to the CEPLC peptide yielded a possible match at residues D115 and L118 (Figure 4B), which are separated by 11.4 angstroms in the structure model of the A33 protein [39]. However, given the results from heptapeptide phage display, we could not rule out an alternative possibility that more complex interactions among D115, Y178, and N125 might influence the shape of the MAb-1G10 epitope by long range hydrogen bonding interactions (Figure 4C).

### Confirmation of critical MAb-1G10 residues by alanine scanning

**Figure 5:**
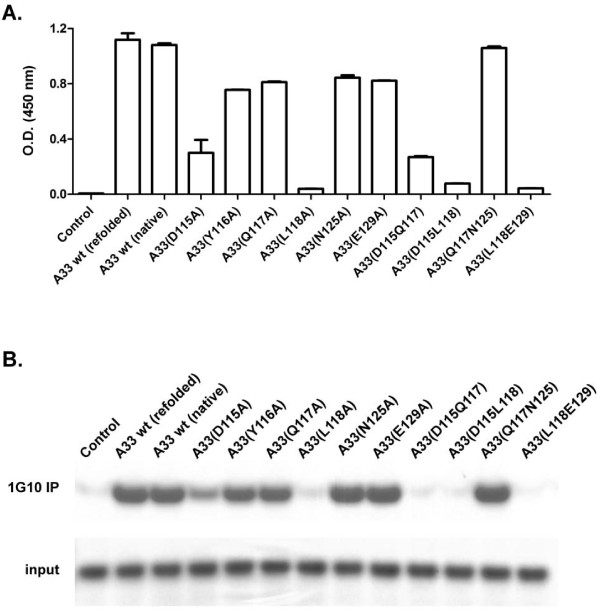
**Binding assays evaluating rA33 proteins containing alanine residues at the specified location(s).** (**A**) rA33 proteins were used in an ELISA format assay to capture MAb-1G10 and were (**B**) tested for their ability to bind MAb-1G10 in an immunoprecipitation assay.

An alanine scanning technique was used to determine if either of these hypotheses regarding the MAb-1G10 epitope structure might be correct. To accomplish this, the ectodomain of wild type A33 (residues 59–186) was expressed in *E. coli* as a His-tagged recombinant protein, isolated from inclusion bodies, and refolded on an affinity purification column. To confirm the process of protein refolding, the native, soluble A33 protein was also generated from *E. coli* cytosolic fractions for comparative purposes. Binding to MAb-1G10 was found comparable by ELISA and immunoprecipitation (Figure 5A and B), as well as to another anti-A33 MAb 10F10 by ELISA [38]. Since protein recovery yields were much higher for the proteins isolated from inclusion bodies, we chose to utilize refolded recombinant proteins for further characterization. We used site directed mutagenesis to prepare a series of A33 variants in which alanine residues were individually substituted for D115, Y116, Q117, L118, N125, and E129. In addition, a series of double alanine substitution A33 variants and a quadruple alanine substitution A33 variant were constructed. All of these were successfully expressed in *E. coli* with similar efficiency and purity as compared to *E. coli* expressed wild type recombinant A33 (rA33; Supplemental Figure 2). MAb-1G10 binding was disrupted by mutations at positions 115 or 118, suggesting that these residues are critical in the MAb-1G10 epitope. In contrast, alanine substitutions at residues 116, 117, 120, 125, and 129 did not prevent interaction with MAb-1G10.

### Confirming the MAb-1G10 epitope using an *in vitro* functional test

**Figure 6:**
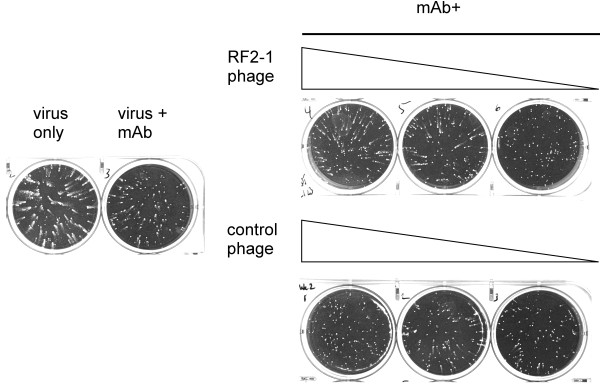
**MAb-1G10 binding phage can interfere with 1G10’s ability to block EEV-mediated virus spread.** Wells labeled ‘MAb+’ received 12.5 μg/mL MAb-1G10 1 hour post infection, and either control phage or phage expressing the RF2-1 consensus sequence at 1 × 10^12^ pfu/mL, 1 × 10^11^ pfu/mL, or 1 × 10^10^ pfu/mL. The monolayers were fixed and stained 46 hours post infection.

**Figure 7:**
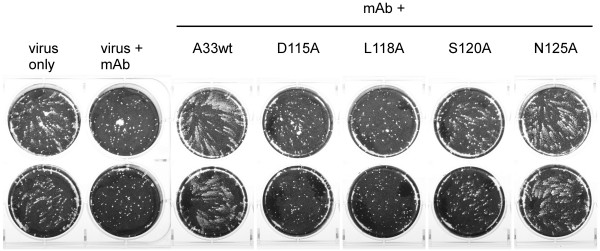
**rA33 proteins containing substitutions at D115 or L118 do not interfere with MAb-1G10’s ability to block EEV-mediated virus spread.** Duplicate wells labeled ‘MAb+’ received 12.5 μg/mL MAb-1G10 1 hour post infection, and a 10-fold molar excess of the indicated rA33 proteins. The monolayers were fixed and stained 46 hours post infection.

Inhibition of EEV spread can be functionally evaluated *in vitro* using an established method in which EEVs released from infected cells rapidly form satellite plaques, commonly referred to as the comet assay. Addition of MAb-1G10 to the supernatant following adsorption of virus to target cells blocked the development of satellite plaques in a dose-dependent manner, with most comets blocked at 12.5 μg/ml (Figure 6). To demonstrate functional relevance of our assays, we tested the ability of our phage and recombinant protein preparations to interfere with the comet neutralizing capacity of MAb-1G10. When phage expressing the CELPC consensus motif (Figure 6) were included in the comet assay along with MAb-1G10 , satellite plaques were restored, demonstrating that MAb-1G10 activity had been abolished. Conversely, when A33 variant proteins containing D115A or L118A mutations were added to the comet assay along with MAb-1G10 , there was no effect on MAb-1G10 comet-neutralizing activity, confirming the loss of a functional MAb-1G10 epitope in these A33 mutant proteins (Figure 7). Addition of Y116A or Q117A variant A33 proteins had no effect on MAb-1G10 activity in the comet assay (data not shown). Interestingly, A33 containing a S120A mutation retained some ability to interact with 1G10 (Figure 7).

## Discussion

We used a randomized peptide library screen to evaluate the A33 comet inhibiting epitope recognized by monoclonal antibody MAb-1G10. Phage technology offers the opportunity to explore the interactive determinants of proteins without preexisting assumptions about the context of the interactions. In this case, the conformationally constrained peptide sequence identified in our library screening was successfully matched with a putative surface-exposed region of vaccinia A33 previously implicated in MAb-1G10 binding. However, our analysis implicated a new upstream residue, D115, in MAb-1G10 binding. As this residue is completely conserved among members of the *Orthopoxvirus* genus, its role in MAb-1G10 binding was not considered in previous studies.

Blocking *in vivo* dissemination of vaccinia virus is an important approach to controlling complications of vaccination in at risk individuals. Poxvirus spread within the host is accelerated by the double-enveloped EEV, which are propelled by actin tails and released prior to target cell lysis [40]. A33 is one of the proteins presented on the EEV surface [41] and deletion of the A33R gene in vaccinia virus reduces disease in an experimental infection model due to inefficient cell-to-cell spread [34]. A33 has also been shown to interact through its cytoplasmic and transmembrane regions with A36 [42], and these EEV proteins together may enhance long-range viral dissemination while limiting superinfection of nearby cells [32]. Vaccine-induced or passively transferred anti-A33 antibodies can mediate protection against lethal orthopoxvirus disease in animal models [31,35-37]. Because A33 is a critical component of vaccinia virulence, neutralizing strategies which target this protein may be particularly effective and thus require appropriate potency assays.

Anti-EEV antibody responses are without exception important for prophylaxis and treatment of poxviruses in animal models. However, serological assessment of anti-EEV antibodies in human smallpox vaccine studies or as a component of passive antibody therapy has been limited. In part this is due to use of well-established PRNT assays, which measure anti-IMV but not anti-EEV activity. Measuring EEV activity has proven challenging due to the fragile nature of the viral envelope [41] and the infectious nature of both EEV and IMV forms of the virus. The comet inhibition assay for EEV antibodies is useful for research studies, but is difficult to validate, and does not provide a robust quantititative result. The importance of measuring anti-EEV antibodies is underscored by observations that anti-B5 and anti-A33 antibody levels are variable in polyclonal VIGIV preparations using research tests (Fisher and Kennedy, unpublished). Binding assays such as ELISAs offer several advantages in terms of reproducibility, speed, and robustness; however to be truly predictive of potency the assay should be specific for a known neutralizing epitope (or epitopes). The current study provides thorough characterization of an A33 conformational comet inhibiting epitope and links the epitope to a viral spread assay. Peptide mimics reflecting the MAb-1G10 binding epitope can be tested in a robust solid phase assay format. Further development and optimization of an assay for evaluation of VIGIV products is currently underway. Additionally, such methods could be used to efficiently screen plasma of vaccinated donors for inclusion in plasma pools used to manufacture VIGIV, or for convalescent plasma intended for therapy in the event of a smallpox outbreak.

To be comprehensive, an optimal anti-EEV assay should include more than one EEV epitope for assessment unless presence of 1G10-like antibodies is shown to be a more general marker for robust anti-EEV responses. A limitation to this broader approach is lack of detailed structural information for other important target EEV proteins such as B5. In the absence of such data, validation of peptides identified in a random display approach is more challenging. Another consideration is accurately reflecting or providing a correlation to effector mechanisms such as complement or Fc receptor involvement [17,43]. Our future studies will include structural analysis of key vaccinia-neutralizing targets to support random peptide library screening efforts, as well as evaluating neutralizing epitope/effector mechanism interactions.

The risks of serious side effects from current live attenuated vaccinia virus vaccines provide the impetus for renewed efforts to develop safer and effective alternatives. So far approaches to develop safe smallpox vaccines have ranged from the study of highly attenuated live vaccinia viruses [44-46] to use of alphavirus replicon vectors expressing vaccinia genes [47] to subunit vaccines delivered either as DNA plasmids [31,48] or purified proteins [49,50]. An alternative approach to vaccine design is the use of molecules that mimic the immunogenic element of interest. For example, peptide mimics coupled with carrier proteins or presented as polymers have been developed for cancer, anti-allergic and contraceptive vaccines [51,52]. Interestingly, peptide mimics need not have similarity to any linear sequence of the antigen but rely on the use of conformation dependent epitopes to stimulate antibodies that will cross-react with the target antigen.

## Conclusions

These results confirm L118 as a component of the MAb-1G10 binding epitope, and further identify D115 as an essential residue. By defining the minimum conformational structure, as well as the conformational arrangement of a short peptide sequence recognized by MAb-1G10, these results introduce the possibility of designing small molecule mimics that may interfere with the function of A33 *in vivo*. Our current study demonstrates how an interdisciplinary approach integrating conformational peptide display, structure mapping, and a biologically relevant assay provides a more complete characterization of an important vaccinia epitope. Our findings suggest an additional option for vaccine subunit development, the possibility of using streamlined assays to assess anti-EEV vaccine responses, and provide a path towards improved potency evaluation of passive immune therapies for human orthopoxvirus disease.

## Methods and materials

### Virus stocks, antibodies and cells

The IHDJ strain of vaccinia virus was obtained from the laboratory of Bernard Moss (NIAID) and passaged once through Vero E6 cells (ATCC). Vero E6 cells were maintained in cDMEM (Dubelco’s minimal essential media containing 10% fetal bovine serum, 4.5 mg/mL D-glucose, 110 μg/mL sodium pyruvate, 100 μM nonessential amino acids, 100 U/mL penicillin/streptomycin, and 0.25 μg/mL amphotericin) and infected at a low MOI (0.2) for 1 hour at 37°C, and were then incubated at 37°C and monitored for cytopathic effect daily. On day 3 postinfection, cells and cell culture supernatant were removed to centrifuge tubes and spun for 15 minutes at 2000 × g at 4°C in an Eppendorf Model 5404R centrifuge equipped with a swinging bucket rotor. The resulting cell pellets were resuspended in cold cDMEM, processed with a chilled Dounce homogenizer, and clarified by centrifugation at 1000 × g for 10 minutes at 4°C. The supernatant was disrupted using a cup horn sonicator for 4 cycles each composed of 30 seconds at 90% power, 50% duty cycle followed by 60 seconds on ice. Aliquots were removed to check sterility and viral titer, and the remainder aliquoted and frozen at -80°C. The MAb-1G10 hybridoma was grown in BD Cell Monoclonal Antibody Medium (Fisher). Hybridoma supernatant was applied to a protein A affinity column (GE Healthcare) and after extensive washes with PBS, the bound antibody was eluted with 0.1M glycine pH 2.5 and immediately neutralized with 1M Tris-HCl (pH 8.0).

### Epitope mapping by randomized peptide phage display library

Selection of peptides from random peptide phage display libraries (PhD-C7C and PhD-12; New England Biolabs, Beverly, MA) was described previously [9]. Briefly, 2 × 10^10^ phages were incubated with MAb-1G10 monoclonal antibody /protein G mixtures for 20 min at room temperature. After eight washings with 0.05 M Tris-HCl buffer (pH 7.5) containing 0.15 M NaCl and 0.05% Tween 20, the phages were eluted from the complex with 0.1 M HCl for 8 min at room temperature and neutralized with 1 M Tris-HCl (pH 9.0). The eluted phages were then amplified in the host strain ER2738 and precipitated with 25% PEG/4 M NaCl. After three additional rounds of selection of amplified phages, DNA from well-separated plaques was sequenced, and the corresponding peptide sequence was then deduced from the DNA sequence.

### Structural analysis

Molecular coordinates for the A33 protein [PDB: 3K7B] used in structural analysis and modeling were obtained from the Protein Data Bank [53] then visualized and analyzed using Pymol (PyMOL Molecular Graphics System, Version 1.1r1, Schrödinger, LLC). To model the structure of the consensus positive phage display peptide, the structural database was queried using BLAST for short sequences containing two cysteines separated by three amino acids (CXXXC). The structures returned from the search were examined for the presence or absence of disulphide bonds and for local conformation. If different the sequence extracted from the database was altered to the sequence of phage display peptide, energy minimized using CHARMM [54] and then compared with the possible locus of the MAb-1G10 epitope.

### Protein expression and purification

Briefly, the DNA sequence encoding the residues from 59 to 186 of wild type A33 was amplified by PCR from vaccinia virus Western Reserve strain [55]. To facilitate protein purification and refolding, a His tag and peptide linker were introduced into the N terminal region of the rA33 construct. Finally, the PCR product was cloned into pET28 (BD Bioscience). Utilizing this construct as a template, plasmids encoding A33 containing the desired mutations were constructed using a kit for site directed mutagenesis (New England Biolabs) according to manufacturer’s instructions using primers described in Table 1. The accuracy of the resulting constructs was verified by DNA sequencing. *E. coli* strain BL21-DE3 was transformed with the resulting plasmids, cultured at 37°C to an OD600 value of approximately 0.4, and then induced with 0.2 mM IPTG for 4 hours. Bacteria were collected by centrifugation for 15 minutes at 5500 × g. The resulting cell pellet was washed with PBS, resuspended in 1 mg/mL lysozyme in PBS, incubated at room temperature for 1 hour, then subjected to sonication on ice for three cycles of 5 minutes each. Alternatively, bacteria were resuspended in 50 mM Tris, 50 mM NaCl, 10 mM EDTA, pH 8.0 and lysed with a French press. Inclusion bodies were collected by centrifugation at 18000 × g for 30 minutes, washed with PBS/0.5% Triton X-100, solubilized overnight in 6 M guanidine, 20 mM Tris, 5 mM DTT, pH 8.0 and then incubated with Ni-NTA agarose beads for 2 hours at room temperature. The beads were loaded onto a Econo-pac column (Biorad) and washed with 3 column volumes of 6 M guanidine. Protein folding was facilitated by washes with a decreasing concentration of guanidine (6 M, 4 M, 2 M, 1 M), and a final wash with PBS. The refolded proteins were eluted from the column with 250 mM inidazole in PBS, pH 8.0 and dialyzed against PBS at 4°C with extensive buffer changes. The protein solution was then clarified by centrifugation at 18000 × g and the resulting supernatant snap-frozen in liquid nitrogen and stored at -80°C.

**Table 1:** **Primers used for recombinant A33 construction by site-directed mutagenesis of the A33R**^**VACV**^**gene**

**Mutation**	**Primer sequence**^**a**^
D115A	ACATTCAGCATACCAGTTATTCTCGGATGCTAAAGCAAATTGCACTGCGG
Y116A	ACATTCAGACGCACAGTTATTCTCGGATGCTAAAGCAAATTGCACTGCGG
Q117A	ACATTCAGACTACGCATTATTCTCGGATGCTAAAGCAAATTGCACTGCGG
L118A	ACATTCAGACTACCAGGCATTCTCGGATGCTAAAGCAAATTGCACTGCGG
S120A	ACATTCAGATTACCAGTTATTCGCTGATGCTAAAGCAAATTGCACTGCGG
N125A	ACATTCAGACTACCAGTTATTCTCGGATGCTAAAGCAGCATGCACTGCGG
E129A	ACATTCAGACTACCAGTTATTCTCGGATGCTAAAGCAAATTGCACTGCGGCATCATCAAC
D115A,Q117A	ACATTCAGCTTACGCATTATTCTCGGATGCTAAAGCAAATTGCACTGCGG
D115A,N125A	ACATTCAGCTTACCAGTTATTCTCGGATGCTAAAGCAGCATGCACTGCGG
Q117A,N125A	ACATTCAGACTACGCATTATTCTCGGATGCTAAAGCAGCATGCACTGCGGAATCATCAAC
L118A,E129A	ACATTCAGACTACCAGGCATTCTCGGATGCTAAAGCAAATTGCACTGCGGCATCATCAAC

^**a**^All primers listed in 5′-3′ direction with the position of the mutation(s) underlined. A common reverse primer was used for all reactions:

AATATATAACAAGAACCCTGGTAATAGAGACCATTACAGCTTTC.

To express and purify soluble recombinant A33 proteins from E.coli, the protein was expressed in BL21(DE3) at 18°C in the presence of 5% glycerol and 2.5% ethanol. The soluble fraction containing A33 was adsorbed onto Talon affinity resin, loaded into an Eco-Pak column and refolded on the column using the method described above. Purity of the proteins was assessed on SDS-PAGE gels (Invitrogen) stained with GelCode Blue (Pierce) or by HPLC analysis with a Zobax GF250 size exclusion column.

### Peptide synthesis

Synthetic phage peptide mimics RF2-1:ACTSYNEPLCGGGSAK-(C6-biotin) and RF2-5:ACGDDWPHECGGGSAK-(C6-biotin) were made by standard 9-fluorenylmethoxy carbonyl chemistry and purified by HPLC (Center for Biologics Evaluation and Research Facility for Biotechnology Resources, US Food and Drug Administration, Rockville, MD). Peptides were confirmed to have the expected molecular weight by matrix-assisted laser desorption ionization-time-of-flight mass spectroscopy. Reduced peptides were generated as previously described [56]. Briefly, the peptide was dissolved in 0.1 M phosphate buffer (pH7.0) and incubated with 20 fold of molar excess of both tris(2-carboxyethyl)phosphine (TCEP) and *N*-Ethylmaleimide (NEM) at room temperature for 2 h. Peptide solutions were stored at -80°C until use.

### ELISAs

96-well polystyrene plates (Corning) were coated with rA33 proteins (10 μg/ml, 100 μl per well) in PBS overnight at 4°C, and unbound rA33 was removed with saline containing 0.5% Tween 20 (PBS-T). Non-specific protein binding was blocked with 5% nonfat dry milk in PBS-T. Serial dilutions of MAb-1G10 in blocking buffer were added to wells and incubated for 1 h at 37°C. Wells were washed 4 times in PBS-T before addition of horseradish peroxidase (HRP) -conjugated anti-mouse secondary antibody (Roche Diagnostics) diluted in blocking buffer. After 1 h incubation, plates were washed 4 times prior to application of soluble HRP substrate (BM Blue, Roche Diagnostics) for 30 min. The reaction was stopped by adding 1M sulfuric acid, and absorbance at 450 nm was determined using a plate reader (Molecular Devices). For detection of antibody binding to biotinylated peptides, peptides diluted in phosphate buffer were added to wells of streptavidin-coated 96-well plates (Thermo Scientific), plates incubated overnight at 4°C, and bound antibody detected as described above.

### Immunoprecipitation analysis

To prepare antibody-conjugated beads, antibody dilutions were incubated with protein A Dynal magnetic beads (Invitrogen) according to manufacturer’s instructions overnight, then treated with excess BS3 crosslinking agent at room temperature for 30 minutes. Crosslinking was quenched by incubating with 1 M Tris buffer. Unbound antibody was removed by incubation with 0.1 M glycine buffer (pH 2.5), followed by three times washing in PBS-T buffer. 5 μg of each rA33 protein was incubated with 200 μl of antibody-conjugated beads for 1 hour at room temperature with consistent rotation. After extensive washing in PBS-T, the bound rA33 proteins were eluted by using 0.1M glycine (pH 2.5). Eluted proteins were analyzed by SDS-PAGE and detected by staining with GelCode Blue (Pierce).

### Comet inhibition assay

Confluent monolayers of Vero E6 cells in six-well cell culture plates (Corning) were infected with the IHD-J strain of VACV at 50–100 pfu per well in 0.2 mL cDMEM. One well was left uninfected as a sham control. After incubation for 1 h at 37°C, the media was removed, and cells were washed twice with 2 mL PBS. Virus-only wells received 2mL cDMEM, and virus + MAb wells received 2 mL cDMEM containing MAb-1G10 at 12.5 μg/mL. Test wells received 2 mL cDMEM containing 12.5 μg/mL MAb-1G10 in combination with concentrations of A33 proteins ranging from 0.1-10 times molar excess over the monoclonal antibody. Some experiments utilized purified phage instead of A33 proteins. Plates were placed in a CO_2_ incubator maintained at 37°C for 46 hours, and comets visualized by staining the monolayers with 0.13% crystal violet in 5% ethanol/3% neutral buffered formalin prior to imaging.

### Bioinformatics and statistical analysis

Data was obtained from the NIAID Virus Pathogen Database and Analysis Resource (ViPR) online through the web site at http://www.viprbrc.org ( http://www.viprbrc.org) and from the Viral Bioinformatics Resource Center (VBRC; http://www.vbrc.org) and was aligned and visualized using multiple sequence alignment tools available at these websites. Statistical analysis was performed using Prism 5 (GraphPad Software). All experiments were performed in duplicate unless otherwise noted.

## Competing interests

The authors declare that they have no competing interests.

## Authors’ contributions

YH and YG generated recombinant A33 proteins, performed ELISA and Western blot analyses. PZ and ES performed random peptide phage mapping and molecular modeling of the resulting peptides. SC performed EEV spread assays and performed ELISAs with the recombinant proteins. JER, MK, and DES participated in the design of the study and provided critical review of the manuscript. RWF conceived the study and participated in its design and coordination. All authors participated in the writing, editing, and final review of the manuscript.
